# From Euglycemia to Recent Onset of Type 2 Diabetes Mellitus: A Proof-of-Concept Study on Circulating microRNA Profiling Reveals Distinct, and Early microRNA Signatures

**DOI:** 10.3390/diagnostics13142443

**Published:** 2023-07-21

**Authors:** Marta Greco, Maria Mirabelli, Alessandro Salatino, Francesca Accattato, Vincenzo Aiello, Francesco S. Brunetti, Eusebio Chiefari, Salvatore A. Pullano, Antonino S. Fiorillo, Daniela P. Foti, Antonio Brunetti

**Affiliations:** 1 Department of Health Sciences, University “Magna Græcia” of Catanzaro, 88100 Catanzaro, Italy; marta.greco@unicz.it (M.G.); maria.mirabelli@unicz.it (M.M.); salatino@unicz.it (A.S.); francescaaccattato@libero.it (F.A.); francescosaverio.brunetti@studenti.unicz.it (F.S.B.); echiefari@unicz.it (E.C.); pullano@unicz.it (S.A.P.); nino@unicz.it (A.S.F.); 2 Department of Precision Medicine, Vanvitelli University, 80133 Naples, Italy; vincenzoaiello91@gmail.com; 3 Department of Experimental and Clinical Medicine, University “Magna Græcia” of Catanzaro, 88100 Catanzaro, Italy

**Keywords:** type 2 diabetes, impaired fasting glucose, circulating miRNAs, biomarkers

## Abstract

Background and aim—Alterations in circulating microRNA (miRNA) expression patterns are thought to be involved in the early stages of prediabetes, as well as in the progression to overt type 2 diabetes mellitus (T2D) and its vascular complications. However, most research findings are conflicting, in part due to differences in miRNA extraction and normalization methods, and in part due to differences in the study populations and their selection. This cross-sectional study seeks to find new potentially useful biomarkers to predict and/or diagnose T2D by investigating the differential expression patterns of circulating miRNAs in the serum of patients with impaired fasting glucose (IFG) and new-onset T2D, with respect to euglycemic controls, using a high-throughput 384-well array and real-time PCR. Methods—Thirty subjects, aged 45–65 years, classified into three matched groups (of 10 participants each) according to their glycometabolic status, namely (1) healthy euglycemic controls, (2) patients with IFG and (3) patients with new-onset, uncomplicated T2D (<2 years since diagnosis) were enrolled. Circulating miRNAs were extracted from blood serum and profiled through real-time PCR on a commercial 384 well-array, containing spotted forward primers for 372 miRNAs. Data analysis was performed by using the online data analysis software GeneGlobe and normalized by the global Ct mean method. Results—Of the 372 analyzed miRNAs, 33 showed a considerably different expression in IFG and new-onset T2D compared to healthy euglycemic controls, with 2 of them down-regulated and 31 up-regulated. Stringent analysis conditions, using a differential fold regulation threshold ≥ 10, revealed that nine miRNAs (hsa-miR-3610, hsa-miR-3200-5p, hsa-miR-4651, hsa-miR-3135b, hsa-miR-1281, hsa-miR-4301, hsa-miR-195-5p, hsa-miR-523-5p and hsa-let-7a-5p) showed a specific increase in new-onset T2D patients compared to IFG patients, suggesting their possible role as early biomarkers of progression from prediabetes to T2D. Moreover, by conventional fold regulation thresholds of ±2, hsa-miR-146a-5p was down-regulated and miR-1225-3p up-regulated in new-onset T2D patients only. Whereas hsa-miR-146a-5p has a well-known role in glucose metabolism, insulin resistance and T2D complications, no association between hsa-miR-1225-3p and T2D has been previously reported. Bioinformatic and computational analysis predict a role of hsa-miR-1225-3p in the pathogenesis of T2D through the interaction with MAP3K1 and HMGA1. Conclusions—The outcomes of this study could aid in the identification and characterization of circulating miRNAs as potential novel biomarkers for the early diagnosis of T2D and may serve as a proof-of-concept for future mechanistic investigations.

## 1. Introduction

MicroRNAs (miRNAs) are a class of short noncoding single-strand RNAs that are generated from genomic DNA. They are characterized by tissue-specific expression patterns and are involved in the regulation of fundamental biological processes, such as tissue development, cell proliferation and differentiation, metabolism, apoptosis and aging, displaying a role in the pathophysiology of diseases [1,2,3,4,5,6,7]. From a biofunctional perspective, miRNAs typically exert a negative effect on gene expression by targeting sites in the 3′ untranslated region (3′UTR) of specific mRNAs, resulting in destabilization, translational repression or mRNA cleavage. However, miRNAs up-regulating gene expression have also been reported [8,9]. To date, more than 1000 miRNAs have been described in humans [10,11], with many of them identifiable in the blood stream [12,13,14,15]. As single-strand RNA molecules, miRNAs are susceptible to degradation by extracellular RNAses, unless they are protected by the encapsulation into lipid-vesicles (i.e., exosomes, microvesicles and apoptotic bodies) [15]. This process preserves the stability of circulating miRNAs, allowing these molecules to act as mediators of intercellular communication [16]. Since their initial discovery, circulating miRNAs have shown specific signatures in response to pathological stimuli, with the potential to be used as biomarkers for diagnosing and/or monitoring diseases [16,17,18]. The interest in circulating miRNAs has increased intensively in recent years, while efficient methods for recovering miRNAs from biological fluids and for quantitative detection, such as real-time PCR and microarray, have become commercially available [15]. With the aid of technical improvements in miRNA profiling, new miRNA molecules have been associated with the dysregulation of many human diseases, including type 2 diabetes (T2D) [16,17,18,19]. T2D can evolve silently over a long period of time, from normal glucose metabolism to impaired fasting glucose (IFG) or prediabetes, and then to overt hyperglycemia and microvascular and macrovascular complications, therefore the scientific community has now shifted its focus on finding strategies for early diagnosis and prevention of T2D, rather than disease control [20,21,22]. Intervening early and specifically with patients who are in the early stages of the disease can help to reduce the high prevalence of T2D and associated risks for adverse clinical outcomes such as blindness, kidney failure, cardiovascular events, stroke and lower limb amputation. This, in turn, would considerably reduce the overall impact of T2D on healthcare [22]. Even though it is described that tissues targeted by insulin release miRNAs into the extracellular space [23,24], and these are involved in the onset of microvascular complications of T2D [15,19,25], there are only few studies investigating the circulating miRNAs signature of the initial stages of disease, when the alterations in glucose metabolism are still mild and clinically asymptomatic [26,27]. Here, we evaluated the expression of circulating miRNAs in age- and sex-matched patients with or without IFG and recent onset of T2D, in order to determine the differences between groups and identify miRNA signatures specifically associated with disease risk. To ensure the accuracy and reliability of our high-throughput miRNA profiling data analysis, we implemented strict criteria for patient enrollment, took careful measures to eliminate any preanalytical biases that could affect miRNA extraction and purification (such as preventing contamination from intracellular miRNAs resulting from hemolysis), and applied a stringent mean-centric method to normalize miRNA expression. This strategy could serve in the identification and characterization of potential novel biomarkers for the early diagnosis of T2D and may represent a proof-of-concept for future mechanistic investigations.

## 2. Materials and Methods

### 2.1. Study Design

For this proof-of-concept, cross-sectional study aimed at discovering novel circulating biomarkers in T2D, 30 unrelated, well-selected, individuals, in the age range of 45–65 years, were enrolled at the Endocrinology Unit “Renato Dulbecco” University Hospital of Catanzaro, Italy. The study population was divided into three age- and sex-matched groups, of 10 patients each, according to their glycometabolic status: (1) euglycemic individuals (control group); (2) patients with impaired fasting glucose (IFG group); (3) patients with a recent (<2 years) diagnosis of T2D (new-onset T2D group), without any micro and macrovascular complications. Diagnosis of IFG and T2D was performed based on 2019 American Diabetes Association Criteria [28]. Inflammation (C-Reactive Protein (CRP) > 6 mg/L; Erythrocyte Sedimentation rate (ESR) > 30 mm/h), obesity (BMI > 30 kg/m^2^), poor glycemic control (glycated hemoglobin, HbA1c > 8%, 64 mmol/mol), smoking, alcohol and drug use, including antidiabetic pharmacological medications with possible impact on miRNAs (i.e., metformin [29]), as well as intercurrent illnesses and moderate-to-severe hypertriglyceridemia (triglycerides > 175 mg/dL, 2.0 mmol/L) were assumed as exclusion criteria. Additionally, in consideration of the possible alterations in miRNA expression throughout the various stages of chronic kidney disease [18,30], to mitigate any potential bias in the detection of circulating miRNAs that are specifically linked to the progression from prediabetes to T2D, individuals with indications of renal impairment (such as an estimated glomerular filtration rate < 60 mL/min/1.73 m^2^ or the presence of microalbuminuria), regardless of whether it was related to diabetic nephropathy [31], were excluded from the study.

### 2.2. Anthropometric and Laboratory Determinations

After 12 h overnight fast, all patients were checked for blood pressure and anthropometric determinations (body weight and height). Venous blood was collected to measure biochemical metabolic parameters, such as fasting glucose, insulin, lipid profile (total, HDL- and LDL-cholesterol and triglycerides) and HbA1c. ESR and CRP were determined to exclude ongoing acute inflammatory processes. In addition, for miRNA testing, venous blood (~10 mL) was collected from each patient in a serum separator tube (Vacutainer R SSTTM, BD, San Jose, CA, USA) and centrifuged within 1 h at 2000× *g* for 15 min at 4 °C. Serum fractions were rapidly aliquoted into cryovial tubes and stored at −80 °C for subsequent miRNA determinations. Measurements of fasting glucose and lipid profile were performed on Cobas 6000 (Roche, Basel, Switzerland). Serum CRP concentrations were determined by immunonephelometric method (Siemens, Alpharetta, GA, USA) using BNII nephelometer (Siemens, Alpharetta, GA, USA). ESR was determined by capillary photometry technology using TEST 1 analyzer (ALIFAX, Padova, Italy). HbA1c was measured by high-performance liquid chromatography using Premier Hb9210™ HbA1c Analyzer (A. Menarini, Firenze, Italy). Serum insulin concentration was measured by the ADVIA Centaur Immunoassay system (Siemens, Alpharetta, GA, USA), using a chemiluminescent immunoassay. All the aforementioned tests were carried out in accordance with the manufacturer’s instructions. Quality control was assessed on a daily basis for all determinations, and the results met the precision targets indicated by the manufacturers.

### 2.3. Circulating miRNAs Profiling

Stored serum aliquots were thawed on ice and centrifuged at 16,000× *g* for 5 min at 4 °C to remove insoluble material. To exclude hemolysis, all samples were subjected to spectrophotometric measurement at 414 nm before continuing with further processing, and, as confirmation test, delta Ct difference between miR-23a-3p and miR-451a had to be less than 5. Circulating miRNAs were then isolated from serum samples (200 µL) of individual patients (*n* = 10 per group) as enriched low-molecular-weight RNA fractions (LMW RNA) using a commercial column-based system (miRNeasy Serum/Plasma Kit^®^, QIAGEN, Hilden, Germany), without specifically isolating miRNA-containing lipid-vesicles. As recommended by the manufacturer, synthetic C. elegans *miR-39* was used as exogenous control and spiked-in to monitor LMW RNA extraction efficiency. The quantity and purity of the extracted LMW RNA (A260/280 ratio = 1.9–2.1 and A260/230 ratio = 2.0–2.2) were determined by a spectrometric method (Nanodrop 1000, Thermo Scientific, Waltham, MA, USA). Afterwards, equal amounts (100 ng) of the individual LMW RNA fractions were pooled for each study group. The pooled LMW RNA fraction (500 ng) was used for a subsequent one-step tailed universal reverse transcription reaction (reaction volume of 100 µL), carried out by miScript II RT Kit (Qiagen) using a thermocycler system (GeneAmp 2700 Thermal Cycler, Applied Biosystems, Waltham, MA, USA). The cDNA products obtained from reverse transcription were used as templates for miRNA profiling by real-time PCR on a high-throughput 384 well-array, containing spotted forward primers for 372 miRNAs with known detectable expression in the human serum (miScript miRNA PCR Array Human Serum & Plasma 384HC^®^, QIAGEN [19] and SYBR Green chemistry (SYBR Green PCR Kit, QIAGEN). Reactions were carried out on QuantStudio™ 12K Flex Real-Time PCR System (Applied Biosystems). miRNAs expression analysis was conducted by a free online software (https://geneglobe.qiagen.com, accessed on 1 February 2023), using 6 reference endogenous controls (small nucleolar RNA: SNORD61, SNORD68, SNORD72, SNORD95, SNORD96A and RNU6B/RNU6-2) and spike-in C. elegans miR-39 as normalizers. MiRNAs whose amplification had reported raw Ct values > 35 were excluded.

### 2.4. Global Ct Mean Normalization and Bioinformatic Analysis

For this large-scale miRNA profiling analysis and biomarker identification, the free online data analysis software GeneGlobe was employed (https://geneglobe.qiagen.com, accessed on 1 February 2023). The Global Ct Mean, which uses the calculated mean expression level of all miRNAs in a given sample, including the reference endogenous controls and spike-in *miR-39*, was automatically implemented as a strongly reliable method of miRNA expression normalization. This normalization strategy assumes that the mean expression level of all miRNAs in a sample, from either the healthy control group or the patient group (IFG or new-onset T2D), is constant when using the same total RNA input [32,33]. By convention, miRNAs with a differential fold regulation > 2 or <−2 between the two groups were considered significant in GeneGlobe analysis, as previously described [34].

To predict the target genes and metabolic phenotypes associated with aberrant circulating miRNAs, a combination of bioinformatic analysis via internet databases and computational tools, including miRbase (http://www.mirbase.org, accessed on 1 February 2023), mirDB (http://Mirdb.org, accessed on 1 February 2023), mirDIP 4.1-integrative database (http://ophid.utoronto.ca/mirDIP, accessed on 1 February 2023), TargetScan (http://www.targetscan.org/vert_71, accessed on 1 February 2023) was used.

### 2.5. Statistical Analysis

Data were expressed as median and interquartile ranges. The Kruskal–Wallis non-parametric test was used to determine intergroup variations in clinical and biochemical parameters. A *p* value less than 0.05 was considered statistically significant. Data analysis was carried out using SPSS, software version 20.0 (SPSS Inc., Chicago, IL, USA).

## 3. Results

### 3.1. Characteristics of the Study Popupulation

The biochemical and anthropometric characteristics of patients enrolled in this study are summarized in Table 1. The three groups under study, namely control, IFG and new-onset T2D, exhibited similar distribution according to gender and age. Additionally, no variations were noted regarding BMI, lipid profile, liver function tests and inflammatory parameters. The sole discrepancy was observed in fasting glucose and HbA1c levels, which accurately reflected the gradual decline in glucose tolerance characteristic of the transition from euglycemia to prediabetes and T2D. This finding confirmed that the selection of cases (IFG and new-onset T2D patients) and controls was appropriate for the purpose of the study. Although diastolic blood pressure levels exhibited a slight, yet statistically significant difference, it did not have clinical implications as none of the patients were considered hypertensive or needed medications to manage their blood pressure (Table 1).

### 3.2. Circulating miRNAs Profiling by Real-Time PCR

**Figure 1:**
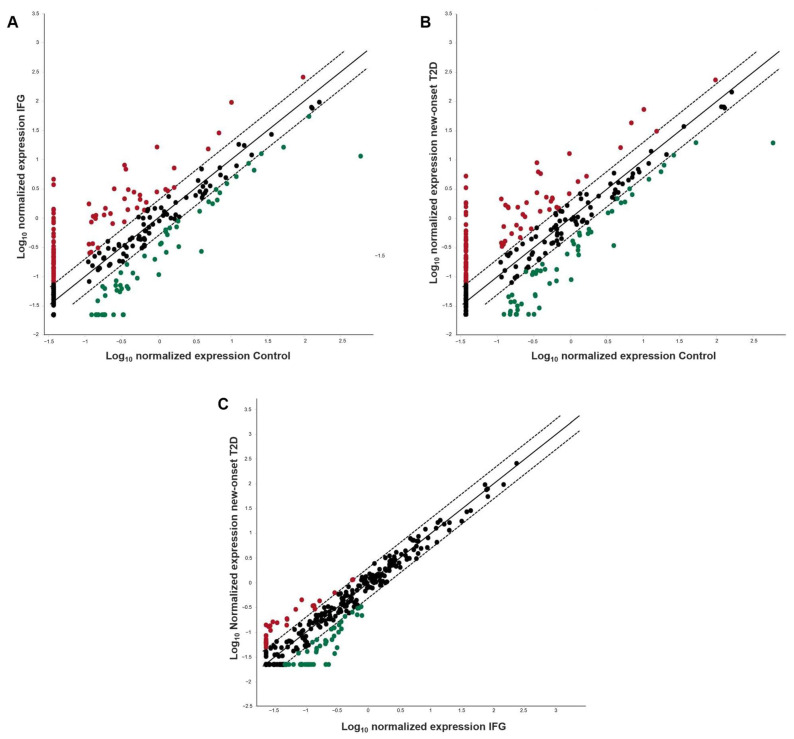
Scatter plot analysis of differential miRNA expression on a logarithmic scale. Red and green dots represent the number of miRNAs that were found to be over-expressed (fold regulation values > 2) and under-expressed (fold regulation values < −2), respectively, in (**A**) IFG vs. control group; (**B**) new-onset T2D vs. control group, (**C**) new-onset T2D vs. IFG group. Black dots represent a lack of differential expression (unvaried miRNAs).

A high-throughput 384 well array profiling of circulating miRNAs was performed on pooled serum LMW RNA fractions, obtained from the three studies and reversely transcribed into cDNAs. In the control group, 3 miRNAs were found to be highly expressed (Ct ≤ 25) and 35 miRNAs were found to be moderately expressed (Ct between 25 and 30). In the IFG group, 4 miRNAs were found to be highly expressed (Ct ≤ 25) and 78 miRNAs were found to be moderately expressed (Ct ≤ 30). In the new-onset T2D group, 7 miRNAs were found to be highly expressed (Ct ≤ 25) and 88 miRNAs were found to be moderately expressed (Ct ≤ 30), therefore suggesting that a progressive change of expression of circulating miRNA could occur in the transition from euglycemia to recent onset of T2D. The supplementary material (Table S1) provides the mature ID of the highly expressed miRNAs for each study group. Out of all miRNAs detectable by real-time PCR (Ct ≤ 35), 95 miRNAs were found to be over-expressed and 54 under-expressed in the IFG group vs. the control group; 109 miRNAs were found to be over-expressed and 52 under-expressed in the new-onset T2D vs. the control group. When comparing new-onset T2D and IFG patients, 49 miRNAs were found to be over-expressed and 33 under-expressed. The scatter plot analysis of differential miRNA expression is illustrated in Figure 1. The mature ID of the differentially expressed miRNAs and their relative quantification (fold regulation) are detailed in Table 2 (for over-expressed miRNAs) and Table 3 (for under-expressed miRNAs).

**Figure 2:**
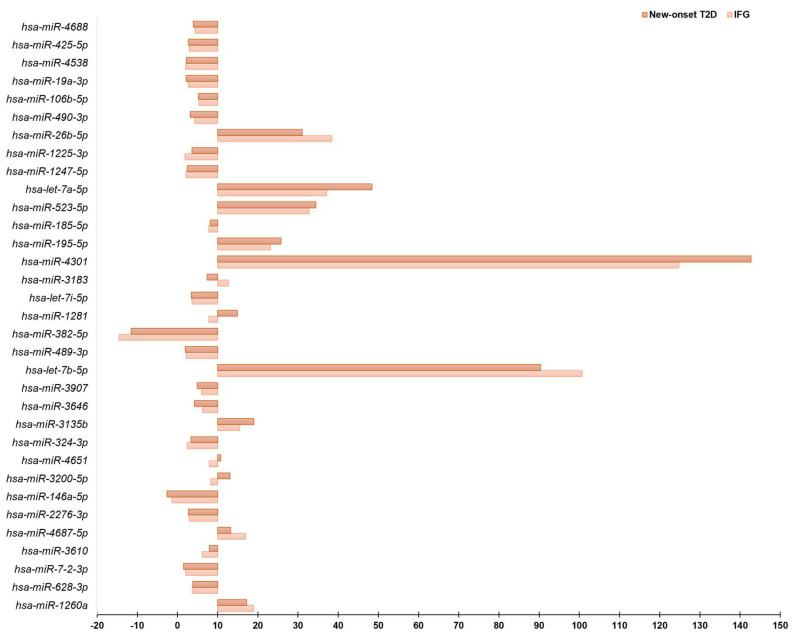
Global Ct mean normalization of miRNA expression and intergroup comparison. The fold regulation values are plotted on the X axis. The Y axis starts at the arbitrary cut-off value of 10 for up-regulation that is considered to be indicative of a significantly different miRNA expression level between both the IFG and new-onset T2D patient groups and the euglycemic control group. Global Ct mean normalization was performed using the GeneGlobe software (https://geneglobe.qiagen.com).

**Figure 3:**
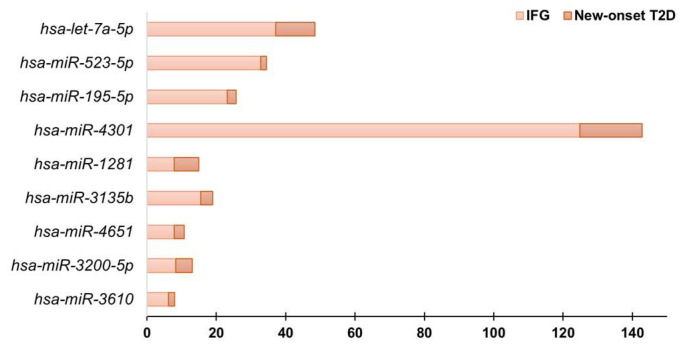
Progressive increase in specific miRNA expression levels in the evolution from IFG to overt T2D with respect to the euglycemic status. The fold regulation values—calculated using the euglycemic control group as reference for both the IFG and new-onset T2D patient groups—are plotted on the X axis. Global Ct mean normalization was performed using the GeneGlobe software (https://geneglobe.qiagen.com).

A total of 33 circulating miRNAs showed considerably different expression levels between patients with IFG or new-onset T2D and matched euglycemic controls (Figure 2). This was determined by using a stringent analysis method (global Ct mean) and considering only those miRNAs whose expression level was relatively low (fold regulation < 10) in either the control or the test group and is reasonably high in the other group (fold regulation ≥ 10). Some miRNAs, such as *hsa-miR-1260a, hsa-miR-4687-5p*, *hsa-miR-3135b*, *hsa-let-7b-5p*, *hsa-let-7a-5p*, *hsa-miR-4301*, *hsa-miR-195-5p*, *hsa-miR-523-5p*, *hsa-miR-26b-5p* were differentially expressed in both IFG and new-onset T2D compared to euglycemic control subjects (Figure 2). Notably, 9 miRNAs (*hsa-miR-3610*, *hsa-miR-3200-5p*, *hsa-miR-4651*, *hsa-miR-3135b*, *hsa-miR-1281*, *hsa-miR-4301*, *hsa-miR-195-5p*, *hsa-miR-523-5p*, *hsa-let-7a-5p*) displayed a significant increase in fold regulation values, calculated using the euglycemic control subjects as the reference group, specifically in patients with new-onset T2D compared to those with IFG (Figure 3). This finding suggests their potential as early biomarkers for disease progression from prediabetes to T2D. Moreover, from a comparative analysis of relative fold regulation data, using the conventional threshold > 2 for up-regulation and <−2 for down-regulation automatically implemented by the Geneglobe software, it was found that *hsa-miR-146a-5p* was considerably down-regulated while *hsa-miR-1225-3p* was up-regulated uniquely in individuals with newly diagnosed T2D in comparison to euglycemic controls (fold regulation in new-onset T2D vs. controls, *hsa-miR-1225-3p*: 3.6; *hsa-miR-146a-5p*: −2.6). These changes in expression levels were not observed in individuals with IFG, despite a similar trend in direction (fold regulation in IFG vs. controls, *hsa-miR-1225-3p*: 1.9; *hsa-miR-146a-5p*: −1.3). Previous research has reported that *hsa-miR-146a-5p* plays a role in glucose metabolism, insulin resistance and development of T2D complications [35,36,37], whereas, to our knowledge, no association between *hsa-miR-1225-3p* and T2D has been formerly described.

## 4. Discussion

Several studies have suggested that circulating miRNAs could be useful for identifying various diseases and predicting clinical outcomes. However, there is no consensus on a miRNA signature that would enable early identification of T2D in its preclinical, asymptomatic stage and prediction of the development of its related complications [15,23].

This proof-of-concept study sought to identify differentially expressed miRNAs in serum of IFG and new-onset T2D patients compared to a control population of healthy euglycemic subjects, in order to evaluate a circulating miRNA signature of the initial onset of T2D. From comparative analysis of high-throughput real-time PCR profiling data, obtained from the total pool of reversely transcribed serum LMW RNA fractions, nine miRNAs, namely *hsa*-*miR-1260a, hsa-miR-4687-5p, hsa-miR-3135b, hsa-let-7b-5p, hsa-let-7a-5p, hsa-miR-4301, hsa-miR-195-5p, hsa-miR-523-5p* and *hsa-miR-26b-5p*, were found over-expressed in both IFG and new-onset T2D patients compared to age- and sex-matched healthy controls. These miRNAs could be used to non-invasively identify patients in the very early stages of the natural history of T2D and strengthen the need for insulin-sensitizing approaches to revert the condition before any complication develops.

Further comparisons revealed that hsa-miR-3610, hsa-miR-3200-5p, hsa-miR-4651, hsa-miR-3135b, hsa-miR-1281, hsa-miR-4301, hsa-miR-195-5p, hsa-miR-523-5p and hsa-let-7a-5p undergo a significant, progressive expression change from euglycemic individuals to patients with IFG and new-onset T2D. This suggests that this circulating miRNA signature can be used as biomarker for progression of disease in those with prediabetes and/or initial laboratory evidence of altered glucose control.

Some of these circulating miRNAs have been shown to be involved in the development of complications of T2D, such as *hsa-miR-1281*, which has been demonstrated by our group, through functional in vitro assays, to have a pathogenic role in diabetic retinopathy, the first microvascular alteration in the natural history of T2D [19]. By targeting *HIF1AN* mRNA, and de-repressing the hypoxia-inducible factor HIF-1a, *hsa-miR-1281* positively regulates the expression of neoangiogenic VEGFA in endothelial cells exposed to a glucose excess environment, therefore contributing to retinal vascular damage [19].

Circulating levels of *hsa-miR-146a-5p* and *hsa-miR-1225-3p* were found, respectively, to be lower and higher, only in the group of new-onset T2D patients. *Hsa-miR-146a-5p* is expressed in white blood cells, and endothelial cells, while it is known to down-regulate the expression of NF-kB and inhibit the NF-kB-induced inflammatory signaling pathways [35,36,37]. A decrease in circulating *hsa-miR-146a-5p* may play an important role in the onset and development of T2D complications through the overexpression of NF-kB and modulation of systemic inflammatory responses that cause glucose control dysfunction [36,37]. In patients with T2D, changes in the blood expression levels of *hsa-miR-146a-5p* have been reported; however, there is conflicting information about whether these changes implicate an increase or decrease [38]. To date, there is no report on *hsa-miR-1225-3p* and its involvement in T2D. However, bioinformatic and computational analysis suggest that this miRNA may be involved in the pathogenesis and complications of T2D through its predicted targets, the mitogen activated protein kinase MAP3K1 and HMGA1, whose transcripts and 3′UTR regions have putative binding sites for *hsa-miR-1225-3p*.

MAP3K1 activates the ERK and JNK kinase cascade in response to various stress stimuli (i.e., cytokines, free fatty acids and hyperglycemia), and appears a crucial mediator in the transition from obesity to T2D [39]. The activation of JNK in liver, adipose tissue and skeletal muscle has been shown to interfere with the transmission of insulin signaling, resulting in insulin resistance. Furthermore, in pancreatic beta cells, this activation results in decreased compensatory insulin secretion [39]. In addition, MAP3K1 activates CHUK and IKBKB, the central protein kinases of the NF-kB pathway [40,41]. MiRNA *hsa-miR-1225-3p* is also predicted to target HMGA1, an architectural transcription factor involved in regulating the expression of several glucose-responsive genes. HMGA1 is known to modulate fundamental biological processes, such as cell proliferation, migration and cell metabolism [29,42,43]. Defective variants of the *HMGA1* gene have been linked to reduced insulin receptor expression and increased susceptibility to T2D [44,45,46]. It is tempting for us to speculate that, by binding to the 3′UTR of the HMGA1 mRNA and facilitating the decay of transcript, increased levels of *hsa-miR-1225-3p* could produce a down-regulation of the insulin receptor expression and insulin resistance.

The bioinformatic analysis of human miRNA target predictions offers potential insights into how other miRNAs that are differentially expressed in our study might interfere with the insulin signaling pathway. For example, *hsa-miR-1260a* might play a role in regulating glucose metabolism and insulin sensitivity, by targeting *PPARGC1A* and *PTPN1*. Decreased expression of PPARG coactivator 1 alpha, encoded by the *PPARGC1A* gene, has been associated with impaired insulin function in skeletal myocytes [47,48,49,50], whereas the tyrosine phosphatase, encoded by the *PTPN1* gene, has been evidenced to act as a negative regulator of the insulin signaling pathway, by dephosphorylating multiple phospho-thyrosine residues within the activated insulin receptor [51,52,53,54]. Moreover, the let-7 family of miRNA has been shown to be involved in the regulation of glucose metabolism and insulin sensitivity by acting on targets associated with the insulin/IGF-1 receptor pathway [55].

MiRNA *hsa-miR-4687-5p*, instead, might be involved in the regulation of TNF-alpha induced NF-kappa-B activation and apoptosis by targeting *ZFAND6* (AN1-type zinc finger protein 6). Persistent hyperglycemia leads to the activation of the NF-kB pathway, which has a key role in the pathogenesis of the micro- and macrovascular complications of T2D. This is caused by an increased expression of different pro-inflammatory cytokines, including TNF-alpha [56,57,58]. TNF-alpha is one of the earliest markers of obesity and T2D, and its raised levels represent a major contributing factor to the development and maintenance of insulin resistance [59,60].

**Figure 4:**
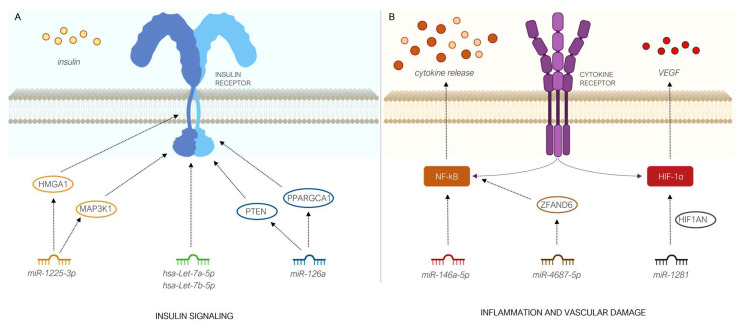
Hypothetical mechanisms by which some of the circulating deregulated miRNAs described in this study may cause insulin resistance (**A**), inflammation and vascular damage (**B**), leading to susceptibility to T2D development. Circulating miRNAs and their predicted target genes are indicated with matched colors.

Figure 4 shows the hypothetic mechanisms emerged from our findings, by which circulating deregulated miRNAs cause insulin resistance, inflammation and vascular damage, both leading to susceptibility to T2D development.

Although there is a growing number of individual miRNA studies focused on the prediction of T2D and its vascular complications, it is important to acknowledge some limitations commonly found in these types of studies. Specifically, individual miRNA-disease association studies often suffer from insufficient statistical power due to small sample sizes, are susceptible to many kinds of experimental biases, and may lead to inconsistent results. Very recently, meta-analytic approaches operating on public high-throughput transcriptomic datasets in the NCBI Gene Expression Omnibus (GEO) repository have identified *hsa-miR-1260a* and *hsa-miR-146a-5p* as two out of nine potential circulating miRNA markers for incident T2D [61]. The findings we have observed in our study are partially congruent with the small signature of miRNAs that has been derived from the aforementioned meta-analysis [61]. However, meta-analytical investigations on circulating miRNAs are unavoidably influenced by the diverse characteristics of available high-throughput miRNA profiling datasets and the unequal quantities of dysregulated miRNAs in T2D patients compared to healthy subjects across different studies. Furthermore, this approach excludes individuals with IFG [61]. A further concern is the considerable diversity in algorithms employed for normalizing miRNAs in the context of disease association studies, encompassing methods such as spike-in normalization, small nucleolar RNA normalization and mean-centric normalization. In light of this, the Global Ct mean normalization has recently undergone validation as the most effective normalization method in high-throughput miRNA profiling studies where the process of miRNA biomarker identification is initiated [31]. The use of diverse normalization techniques in high-throughput miRNA profiling studies may explain the lack of prior reports indicating an association between T2D and the increased expression of circulating *hsa-miR-1225-3p* [62]. With the broader implementation of the Global Ct mean normalization method in high-throughput miRNA profiling studies, it is conceivable that greater reproducibility will be achieved, particularly in the systematic identification of circulating miRNA markers that are candidate for further validation processes. In this regard, it is noteworthy to mention that in our study, the differential expression of circulating miRNAs and the direction of variation remained consistent even when alternative normalization methods, such as those based on small nucleolar RNAs, were used. Nevertheless, there was a discrepancy in the magnitude of fold regulation observed, as normalizing with the Global Ct mean yielded lower values compared to less stringent normalization methods.

Although the mechanisms underlying changes in circulating miRNA levels in T2D are not fully understood, previous studies have suggested that miRNAs can be actively or passively released by a variety of cells and, by the association with microvesicles or protein complexes, transferred in an active form to recipient cells [15]. Based on the present findings, although the source and target cells of extracellular miRNAs remain uncertain, as well as their specific carriers [63], we can hypothesize that modifications in specific circulating miRNAs, potentially with a pathogenic role, may occur simultaneously during the course of disease progression from normal glucose tolerance to IFG and overt T2D. While the main strength of this work is the very strict selection of the study population, in which both clinical confounders and pharmacological therapies were excluded, and the technical rigor in the procedures applied to blood samples, the major limitation of the study is the lack of individual validation of the identified circulating miRNA signature in a separate cohort of patients with new-onset T2D and prediabetes. The design of this work, which involves the high-throughput profiling of pooled LMW RNA fractions from participants, limits the potential for conducting correlation analysis between circulating miRNAs and conventional clinical and laboratory parameters. Additionally, it hinders the identification of the precise miRNA-regulated network responsible for these disease traits. However, this study was designed as a proof-of-concept study that can now encourage new research on selected miRNAs, including the novel *hsa-miR-1225-3p*, in the pathogenesis and therapeutics of T2D.

## 5. Conclusions

Despite intensive research and rapid progress in recent decades, the key mechanisms underlying T2D pathogenesis, and the development of complications are still not fully elucidated. Circulating miRNAs can be used as noninvasive biomarkers for the identification and monitoring of various diseases, including T2D. High-throughput profiling data analysis revealed that some miRNAs could be considered as noninvasive diagnostic biomarkers of the recent onset of T2D *(hsa-miR-1260a, hsa-miR-4687-5p, hsa-miR-3135b, hsa-let-7b-5p and hsa-let-7a-5p, hsa-miR-4301, hsa-miR-195-5p, hsa-miR-523-5p* and *hsa-miR-26b-5p*) while others (*hsa-miR-3610, hsa-miR-3200-5p* and *hsa-miR-4651, hsa-miR-3135b, hsa-miR-1281, hsa-miR-4301, hsa-miR-195-5p, hsa-miR-523-5p* and *hsa-let-7a-5p*) as biomarkers of disease progression from prediabetes to T2D. For the first time in this study, it is suggested that the up-regulation of *hsa-miR-1225-3p* may be responsible for insulin resistance in T2D patients.

## Figures and Tables

**Table 1:** General characteristics and laboratory parameters of study participants.

	Control (*n* = 10)	IFG (*n* = 10)	New-Onset T2D (*n* = 10)	*p* Value
Female sex (%)	6 (60%)	6 (60%)	6 (60%)	-
Age (yrs)	53.50 (47–58)	51.50 (47–61)	58 (57–65)	0.167
BMI (kg/m^2^)	26 (22–28)	28 (25–30)	29 (27–30)	0.060
SBP (mmHg)	120 (119–120)	120 (120–130)	120 (120–125)	0.170
DBP (mmHg)	80 (78–80)	85 (80–85)	80 (80–85)	**0.048**
Fasting glucose (mg/dL)	88 (83–91)	113 (105–118)	135 (118–165)	**<0.001**
HbA1c (%)	5.10 (4.98–5.38)	5.70 (5.43–6.10)	6.20 (6.0–6.60)	**<0.001**
Total cholesterol (mg/dL)	179 (160–200)	204 (178–223)	185 (165–210)	0.337
LDL-cholesterol (mg/dL)	110 (93–125)	142 (109–158)	113 (103–125)	0.133
HDL-cholesterol (mg/dL)	57 (44–60)	42 (37–56)	55 (40–56)	0.207
Triglycerides (mg/dL)	88 (75–127)	103 (67–158)	120 (93–144)	0.480
CRP (mg/L)	3.16 (3.16–3.36)	3.45 (3.16–10)	4.08 (0.64–5.75)	0.659
ESR (mm/h)	6.50 (4.75–14.25)	9.50 (3.75–28.25)	9.0 (5.0–10)	0.806
T2D duration (yrs)	-	-	2 (0–2)	-

Data are expressed as medians and interquartile ranges (IQR). The inter-group variability is determined by Kruskal–Wallis test. Bold values denote statistical significance at *p* < 0.05. BMI, Body Mass Index; SBP, Systolic Blood Pressure; DBP, Diastolic Blood Pressure; HbA1c, Glycated hemoglobin; ESR, Erythrocyte Sedimentation Rate; CRP, C Reactive Protein.

**Table 2:** Over-expressed miRNAs in IFG vs. control group, new-onset T2D vs. control group and new-onset T2D vs. IFG group.

IFG vs. Control		New-Onset T2D vs. Control		New-Onset T2D vs. IFG	
Mature ID	Fold Regulation	Mature ID	Fold Regulation	Mature ID	Fold Regulation
hsa-miR-4301	124.8	hsa-miR-4301	142.74	hsa-miR-4732-3p	10.29
hsa-let-7b-5p	100.66	hsa-let-7b-5p	90.34	hsa-miR-142-5p	9.28
hsa-miR-26b-5p	38.41	hsa-miR-1287-5p	50.12	hsa-miR-877-3p	7.75
hsa-let-7a-5p	37.1	hsa-let-7a-5p	48.41	hsa-miR-3141	6.43
hsa-miR-523-5p	32.75	hsa-miR-1913	42.73	hsa-miR-2467-3p	5.91
hsa-miR-1287-5p	26.97	hsa-miR-523-5p	34.47	hsa-miR-7-5p	5.52
hsa-miR-1913	24.14	hsa-miR-26b-5p	31.06	hsa-miR-324-5p	5.11
hsa-miR-195-5p	23.16	hsa-miR-195-5p	25.76	hsa-miR-942-5p	4.87
hsa-miR-100-5p	22.37	hsa-miR-1587	21.07	hsa-miR-550a-5p	4.64
hsa-miR-1260a	18.94	hsa-miR-3135b	18.99	hsa-miR-10b-5p	4.33
hsa-miR-365b-3p	17.43	hsa-miR-1260a	17.23	hsa-miR-361-3p	3.87
hsa-miR-4687-5p	16.95	hsa-miR-1260b	16.53	hsa-miR-2110	3.85
hsa-miR-3135b	15.49	hsa-miR-20b-5p	15.97	hsa-miR-548o-5p	3.79
hsa-let-7f-5p	14.66	hsa-miR-1281	14.9	hsa-miR-331-3p	3.74
hsa-miR-3131	13.12	hsa-miR-3131	14.49	hsa-miR-769-5p	3.66
hsa-miR-3183	12.67	hsa-miR-4687-5p	13.24	hsa-miR-18a-5p	3.66
hsa-miR-20b-5p	10.8	hsa-miR-3200-5p	13.06	hsa-miR-3191-3p	3.64
hsa-miR-1260b	10.44	hsa-miR-100-5p	12.88	hsa-miR-140-5p	3.61
hsa-miR-223-3p	9.4	hsa-let-7c-5p	11.94	hsa-miR-1587	3.59
hsa-miR-370-3p	8.9	hsa-miR-365b-3p	11.77	hsa-miR-130b-3p	3.49
hsa-miR-3200-5p	8.3	hsa-let-7f-5p	11.37	hsa-miR-99a-5p	3.35
hsa-miR-3911	8.02	hsa-miR-671-3p	11.29	hsa-miR-3185	3.33
hsa-miR-4651	7.91	hsa-miR-625-3p	11.29	hsa-miR-596	3.3
hsa-miR-1281	7.85	hsa-miR-4651	10.76	hsa-miR-181c-5p	3.26
hsa-miR-378g	7.85	hsa-miR-4505	9.9	hsa-miR-138-1-3p	3.17
hsa-miR-30e-3p	7.8	hsa-miR-144-3p	9.76	hsa-miR-4505	3.15
hsa-miR-185-5p	7.75	hsa-miR-1237-3p	9.56	hsa-miR-1539	3.1
hsa-miR-1237-3p	7.59	hsa-miR-378g	9.36	hsa-miR-29b-3p	3
hsa-let-7c-5p	7.23	hsa-miR-1183	9.11	hsa-miR-193a-5p	2.92
hsa-miR-144-3p	7.08	hsa-miR-596	8.92	hsa-miR-376c-3p	2.82
hsa-miR-671-3p	7.08	hsa-miR-1910-5p	8.86	hsa-miR-3176	2.56
hsa-miR-101-3p	7.03	hsa-miR-3141	8.44	hsa-miR-1183	2.4
hsa-miR-1301-3p	6.88	hsa-miR-185-5p	8.15	hsa-miR-501-5p	2.38
hsa-miR-1910-5p	6.7	hsa-miR-3610	7.93	hsa-miR-574-3p	2.32
hsa-miR-1290	6.38	hsa-miR-877-3p	7.71	hsa-miR-1207-5p	2.3
hsa-miR-3646	6.2	hsa-miR-1207-5p	7.61	hsa-miR-28-3p	2.27
hsa-miR-3610	6.16	hsa-miR-3911	7.55	hsa-miR-874-3p	2.24
hsa-miR-4291	5.95	hsa-miR-99a-5p	7.55	hsa-miR-92b-3p	2.19
hsa-miR-3907	5.95	hsa-miR-3183	7.4	hsa-miR-361-5p	2.19
hsa-miR-1203	5.95	hsa-let-7e-5p	7.35	hsa-miR-3622a-5p	2.18
hsa-miR-1587	5.87	hsa-miR-223-3p	7.2	hsa-miR-339-3p	2.16
hsa-miR-4732-5p	5.79	hsa-miR-373-5p	6.31	hsa-miR-375	2.16
hsa-miR-625-3p	5.63	hsa-miR-4274	6.18	hsa-miR-4306	2.13
hsa-miR-106b-5p	5.36	hsa-miR-370-3p	6.05	hsa-miR-154-5p	2.12
hsa-miR-181c-3p	5.25	hsa-miR-101-3p	5.93	hsa-miR-199b-5p	2.1
hsa-miR-18a-3p	5.08	hsa-miR-181c-5p	5.69	hsa-miR-454-3p	2.08
hsa-miR-4274	5.01	hsa-miR-4689	5.65	hsa-miR-1976	2.02
hsa-miR-219a-1-3p	4.8	hsa-miR-142-5p	5.57	hsa-miR-378b	2.01
hsa-let-7e-5p	4.74	hsa-miR-1301-3p	5.53	hsa-miR-625-3p	2.01
hsa-miR-34c-3p	4.54	hsa-miR-106b-5p	5.23		
hsa-miR-877-5p	4.51	hsa-miR-3907	4.92		
hsa-miR-1909-5p	4.48	hsa-miR-1290	4.81		
hsa-miR-146b-5p	4.45	hsa-miR-877-5p	4.71		
hsa-miR-4688	4.39	hsa-miR-1909-5p	4.68		
hsa-miR-363-3p	4.39	hsa-miR-4732-5p	4.68		
hsa-miR-490-3p	4.33	hsa-miR-320e	4.52		
hsa-miR-373-5p	4.18	hsa-miR-138-1-3p	4.49		
hsa-miR-4689	3.93	hsa-let-7d-5p	4.46		
hsa-miR-4267	3.85	hsa-miR-10a-5p	4.25		
hsa-miR-1183	3.79	hsa-miR-1203	4.25		
hsa-miR-629-5p	3.77	hsa-miR-3646	4.19		
hsa-miR-10a-5p	3.69	hsa-miR-34c-3p	4.05		
hsa-miR-628-3p	3.66	hsa-miR-181c-3p	4.02		
hsa-miR-345-5p	3.64	hsa-miR-1539	3.96		
hsa-let-7i-5p	3.61	hsa-miR-3185	3.96		
hsa-miR-320e	3.4	hsa-miR-4688	3.96		
hsa-let-7d-5p	3.37	hsa-miR-3191-3p	3.91		
hsa-miR-1207-5p	3.3	hsa-miR-4291	3.88		
hsa-miR-27b-3p	3.17	hsa-miR-363-3p	3.88		
hsa-miR-4505	3.15	hsa-miR-219a-1-3p	3.83		
hsa-miR-4516	3.12	hsa-miR-628-3p	3.8		
hsa-miR-330-3p	3.06	hsa-miR-1225-3p	3.62		
hsa-miR-487b-3p	2.94	hsa-miR-2467-3p	3.55		
hsa-miR-425-5p	2.91	hsa-miR-375	3.5		
hsa-miR-2276-3p	2.89	hsa-let-7i-5p	3.43		
hsa-miR-675-3p	2.87	hsa-miR-324-3p	3.36		
hsa-miR-605-5p	2.78	hsa-miR-27b-3p	3.36		
hsa-miR-596	2.7	hsa-miR-4516	3.33		
hsa-miR-19a-3p	2.7	hsa-miR-92b-3p	3.2		
hsa-miR-451a	2.65	hsa-miR-675-3p	3.2		
hsa-miR-7-1-3p	2.54	hsa-miR-490-3p	3.15		
hsa-miR-127-3p	2.5	hsa-miR-146b-5p	3.09		
hsa-miR-324-3p	2.43	hsa-miR-4267	2.98		
hsa-miR-193b-3p	2.33	hsa-miR-425-3p	2.96		
hsa-miR-378a-5p	2.3	hsa-miR-942-5p	2.92		
hsa-miR-99a-5p	2.26	hsa-miR-374c-5p	2.86		
hsa-miR-374c-5p	2.19	hsa-miR-3176	2.8		
hsa-miR-425-3p	2.16	hsa-miR-550a-5p	2.78		
hsa-miR-489-3p	2.15	hsa-miR-2276-3p	2.75		
hsa-miR-1247-5p	2.12	hsa-miR-425-5p	2.75		
hsa-miR-188-5p	2.1	hsa-miR-190a-5p	2.67		
hsa-miR-4422	2.06	hsa-miR-10b-5p	2.6		
hsa-miR-664a-3p	2.06	hsa-miR-139-3p	2.58		
hsa-miR-7-2-3p	2	hsa-miR-188-5p	2.46		
hsa-miR-4538	2	hsa-miR-451a	2.41		
		hsa-miR-1247-5p	2.39		
		hsa-miR-664a-3p	2.37		
		hsa-miR-361-3p	2.32		
		hsa-miR-2110	2.31		
		hsa-miR-605-5p	2.29		
		hsa-miR-4302	2.29		
		hsa-miR-1180-3p	2.28		
		hsa-miR-548o-5p	2.28		
		hsa-miR-193b-3p	2.28		
		hsa-miR-331-3p	2.25		
		hsa-miR-7-1-3p	2.25		
		hsa-miR-769-5p	2.2		
		hsa-miR-4538	2.18		
		hsa-miR-140-5p	2.17		
		hsa-miR-19a-3p	2.17		
		hsa-miR-378b	2.04		
		hsa-miR-4454	2.04		

**Table 3:** Under-expressed miRNAs in IFG vs. control group, new-onset T2D vs. control group and new-onset T2D vs. IFG group.

IFG vs. Control		New-Onset T2D vs. Control		New-Onset T2D vs. IFG	
Mature ID	Fold Regulation	Mature ID	Fold Regulation	Mature ID	Fold Regulation
hsa-miR-328-3p	−52.22	hsa-miR-328-3p	−30.54	hsa-miR-629-5p	−6.13
hsa-miR-324-5p	−15.1	hsa-miR-598-3p	−13.96	hsa-miR-152-3p	−5.53
hsa-miR-154-5p	−14.89	hsa-miR-152-3p	−12.84	hsa-miR-1307-3p	−5.3
hsa-miR-382-5p	−14.59	hsa-miR-502-3p	−11.74	hsa-miR-3651	−5.23
hsa-miR-7-5p	−11.05	hsa-miR-382-5p	−11.49	hsa-miR-345-5p	−5.08
hsa-miR-409-3p	−9.49	hsa-miR-409-3p	−11.49	hsa-miR-4323	−4.52
hsa-miR-210-3p	−8.44	hsa-miR-181d-5p	−8.07	hsa-miR-30e-3p	−4.25
hsa-miR-181d-5p	−8.26	hsa-miR-28-5p	−7.91	hsa-miR-487b-3p	−4.05
hsa-miR-28-5p	−8.09	hsa-miR-1307-3p	−7.58	hsa-miR-18a-3p	−3.83
hsa-miR-4258	−7.71	hsa-miR-154−5p	-7.03	hsa-miR-378a-5p	−3.75
hsa-miR-340-5p	−6.85	hsa-miR-424-3p	−6.98	hsa-miR-502-3p	−3.72
hsa-miR-627-5p	−6.8	hsa-miR-132-3p	−6.79	hsa-miR-181a-5p	−3.67
hsa-miR-4732-3p	−6.35	hsa-miR-181a-5p	−6.65	hsa-miR-378e	−3.2
hsa-miR-1976	−6.26	hsa-miR-30a-3p	−6.56	hsa-miR-590-5p	−3
hsa-miR-151a-5p	−5.92	hsa-miR-4323	−6.25	hsa-miR-421	−2.84
hsa-miR-34a-5p	−5.53	hsa-miR-3651	−6.03	hsa-miR-301a-3p	−2.84
hsa-miR-29b-3p	−5.53	hsa-miR-210-3p	−5.91	hsa-miR-206	−2.74
hsa-miR-132-3p	−5.53	hsa-miR-627-5p	−5.87	hsa-let-7a-3p	−2.72
hsa-miR-224-5p	−5.49	hsa-miR-339-5p	−5.47	hsa-miR-598-3p	−2.72
hsa-miR-598-3p	−5.12	hsa-miR-34a-5p	−5.4	hsa-miR-374a-5p	−2.72
hsa-miR-339-5p	−4.98	hsa-miR-199a-5p	−4.87	hsa-miR-26b-3p	−2.67
hsa-miR-130b-3p	−4.68	hsa-miR-4258	−4.57	hsa-miR-192-5p	−2.67
hsa-miR-130a-3p	−4.58	hsa-miR-378a-3p	−4.45	hsa-miR-505-3p	−2.61
hsa-miR-18a-5p	−4.31	hsa-miR-340-5p	−4.12	hsa-miR-199a-5p	−2.6
hsa-miR-424-3p	−3.99	hsa-miR-93-3p	−3.87	hsa-miR-3923	−2.58
hsa-miR-361-5p	−3.94	hsa-miR-130a-3p	−3.69	hsa-miR-3653-3p	−2.51
hsa-miR-30a-3p	−3.88	hsa-miR-15a-5p	−3.35	hsa-miR-15a-5p	−2.49
hsa-miR-199b-5p	−3.75	hsa-miR-151a-5p	−3.3	hsa-miR-1-3p	−2.42
hsa-miR-16-5p	−3.22	hsa-miR-421	−3.26	hsa-miR-378a-3p	−2.2
hsa-miR-342-3p	−3.17	hsa-miR-199b-3p	−3.19	hsa-miR-485-5p	−2.15
hsa-miR-93-5p	−3.17	hsa-miR-1976	−3.1	hsa-miR-338-3p	−2.12
hsa-miR-150-5p	−3.15	hsa-miR-194-5p	−3.06	hsa-miR-199b-3p	−2.08
hsa-miR-502-3p	−3.15	hsa-miR-324-5p	−2.95	hsa-miR-151b	−2.05
hsa-miR-194-5p	−2.98	hsa-miR-93-5p	−2.93		
hsa-miR-5095	−2.98	hsa-miR-125a-5p	−2.81		
hsa-miR-193a-5p	−2.9	hsa-miR-191-5p	−2.78		
hsa-miR-125a-5p	−2.84	hsa-miR-224-5p	−2.76		
hsa-miR-433-3p	−2.65	hsa-miR-151a-3p	−2.76		
hsa-miR-24-3p	−2.6	hsa-miR-24-3p	−2.76		
hsa-miR-19b-3p	−2.6	hsa-miR-192-5p	−2.74		
hsa-miR-186-5p	−2.58	hsa-miR-19b-3p	−2.68		
hsa-miR-130b-5p	−2.56	hsa-miR-16-5p	−2.66		
hsa-miR-151a-3p	−2.54	hsa-miR-146a-5p	−2.64		
hsa-miR-152-3p	−2.32	hsa-miR-186-5p	−2.61		
hsa-miR-484	−2.31	hsa-miR−433-3p	−2.59		
hsa-miR-320a	−2.26	hsa-miR-126-3p	−2.55		
hsa-miR-93-3p	−2.2	hsa-miR-505-3p	−2.52		
hsa-let-7g-5p	−2.14	hsa-miR-21-5p	−2.37		
hsa-miR-92a-3p	−2.14	hsa-miR-151b	−2.35		
hsa-let-7d-3p	−2.12	hsa-miR-25-3p	−2.16		
hsa-miR-25-3p	−2.07	hsa-miR-342-3p	−2.02		
hsa-miR-423-5p	−2.02	hsa-miR-15b-5p	−2.02		
hsa-miR-191-5p	−2.02	hsa-miR-7-5p	−2		
hsa-miR-378a-3p	−2.02				

## Data Availability

Data supporting the reported results are available from the corresponding author upon reasonable request.

## Supplementary Materials

The following supporting information can be downloaded at: https://www.mdpi.com/article/10.3390/diagnostics13142443/s1, Table S1: Highly expressed miRNAs in euglycemic controls, and IFG and new-onset T2D patients.
