# Supplementary material for: From Euglycemia to Recent Onset of Type 2 Diabetes Mellitus: A Proof-of-Concept Study on Circulating microRNA Profiling Reveals Distinct, and Early microRNA Signatures
Source: Diagnostics (Basel). 2023 Jul 21;13(14):2443. doi: 10.3390/diagnostics13142443 (PMC10377827; doi:10.3390/diagnostics13142443)
Supplement: Supplementary file 1 [file diagnostics-13-02443-s001.zip › diagnostics-2476629-supplementary.pdf]

Supplementary Material

From euglycemia to recent onset of Type 2 diabetes mellitus: circulating microRNA profiling reveals distinct, and early microRNA signatures

Marta Greco, Maria Mirabelli, Alessandro Salatino, Francesca Accattato, Vincenzo Aiello, Francesco S. Brunetti, Eusebio Chiefari, Salvatore A. Pullano, Antonino S. Fiorillo, Daniela P. Foti and Antonio Brunetti

Table S1. Highly expressed miRNAs (Ct ≤ 25) in euglycemic controls, and IFG and new-onset T2D patients.

**Control**		**IFG**		**New-onset T2D**	
Mature ID	Ct	Mature ID	Ct	Mature ID	Ct
hsa-miR-328-3p	22.58	*hsa-miR-451a*	22.04	*hsa-miR-451a*	21.68
hsa-miR-486-5p	24.46	*hsa-miR-486-5p*	23.46	*hsa-miR-486-5p*	22.36
hsa-miR-92a-3p	24.93	*hsa-miR-223-3p*	23.47	*hsa-miR-92a-3p*	23.20
		hsa-miR-92a-3p	24.27	*hsa-miR-223-3p*	23.35
				hsa-miR-373-5p	24.11
				hsa-miR-1280	24.32
				hsa-miR-4454	24.59
